# Artificial intelligence models utilize lifestyle factors to predict dry eye related outcomes

**DOI:** 10.1038/s41598-025-96778-x

**Published:** 2025-04-18

**Authors:** Andrew D. Graham, Jiayun Wang, Tejasvi Kothapalli, Jennifer E. Ding, Helen Tasho, Alisa Molina, Vivien Tse, Sarah M. Chang, Stella X. Yu, Meng C. Lin

**Affiliations:** 1 Vision Science Group, University of California, Berkeley, USA; 2 Clinical Research Center, School of Optometry, University of California, 360 Minor Hall, Berkeley, CA 94720 - 2020 USA; 3 Department of Electrical Engineering and Computer Science, University of California, Berkeley, USA; 4 International Computer Science Institute, Berkeley, USA; 5 Electrical Engineering and Computer Science Department, University of Michigan, Ann Arbor, USA

**Keywords:** Dry eye, Meibomian gland dysfunction, Lifestyle, Artificial intelligence, Machine learning, Age, Contact lens wear, Alcohol, Driving, Exercise, Near work, Airplane cabin, Outdoor exposure, Blepharitis, Line of Marx, Eyelid notching, Tear film instability, Medical research, Outcomes research, Translational research, Eye diseases, Conjunctival diseases, Corneal diseases, Eyelid diseases, Vision disorders

## Abstract

The purpose of this study is to examine and interpret machine learning models that predict dry eye (DE)-related clinical signs, subjective symptoms, and clinician diagnoses by heavily weighting lifestyle factors in the predictions. Machine learning models were trained to take clinical assessments of the ocular surface, eyelids, and tear film, combined with symptom scores from validated questionnaire instruments for DE and clinician diagnoses of ocular surface diseases, and perform a classification into DE-related outcome categories. Outcomes are presented for which the data-driven algorithm identified subject characteristics, lifestyle, behaviors, or environmental exposures as heavily weighted predictors. Models were assessed by 5-fold cross-validation accuracy and class-wise statistics of the predictors. Age was a heavily weighted factor in predictions of eyelid notching, Line of Marx anterior displacement, and fluorescein tear breakup time (FTBUT), as well as visual analog scale symptom ratings and a clinician diagnosis of blepharitis. Comfortable contact lens wearing time was heavily weighted in predictions of DE symptom ratings. Time spent in near work, alcohol consumption, exercise, and time spent outdoors were heavily weighted predictors for several ocular signs and symptoms. Exposure to airplane cabin environments and driving a car were predictors of DE-related symptoms but not clinical signs. Prediction accuracies for DE-related symptoms ranged from 60.7 to 86.5%, for diagnoses from 73.7 to 80.1%, and for clinical signs from 66.9 to 98.7%. The results emphasize the importance of lifestyle, subject, and environmental characteristics in the etiology of ocular surface disease. Lifestyle factors should be taken into account in clinical research and care to a far greater extent than has been the case to date.

**Supplementary Information:**

The online version contains supplementary material available at 10.1038/s41598-025-96778-x.

## Introduction

In the study of dry eye (DE), patient characteristics, lifestyle behaviors, and risk exposures have recently emerged as critical to its etiology and to its diagnosis, treatment and management. While the vast literature on DE and related ocular surface diseases has tended to focus on mechanisms of pathology, development of diagnostic instruments both objective and subjective, and on treatment and management, lifestyle factors have historically been secondary to most analyses, when they are included at all. Recently, the Tear Film and Ocular Surface Society (TFOS) workshop report described ocular surface disease as a “lifestyle epidemic”^1^, and interest in the impact of patient lifestyle and behaviors is receiving renewed and much needed attention.

In recent years, artificial intelligence has proven to be a valuable tool in biomedical research and health care, however the use of this technology in the study and management of ocular surface diseases like DE has lagged behind its use in other aspects of vision such as retinal imaging^2^. One area of nascent advancement has been the detailed analysis of Meibomian gland morphology from infrared imaging of the everted eyelids, known as meibography^3^. Recent work has demonstrated the ability to use machine learning models to quantify Meibomian gland morphological characteristics from meibography imaging^4,5^, and to combine the imaging results with patient lifestyle and behavioral factors, clinical measurements, symptomatological assessments, and clinician diagnoses to predict outcomes related to Meibomian gland dysfunction (MGD), DE, and other ocular surface pathology^6^.

When the most heavily weighted variables used by machine learning models to predict DE-related outcomes are examined, many subject characteristics, lifestyle qualities, behavioral factors, and associated environmental exposures play a prominent role. These emerging artificial intelligence models can facilitate the discovery of novel relationships among clinical, lifestyle, and symptom variables, allow examination of previously determined relationships from a new perspective, and generate new hypotheses for further investigation^7,8^. The importance of lifestyle factors in machine learning model predictions of ocular surface disease-related outcomes is the focus of the current work.

## Methods

Subjects 18 years of age or older with no history of ocular surgery, no active ocular infections, and not currently taking medications known to affect the anterior eye, eyelids or tear film were eligible for the study. Both contact lens wearers and non-wearers were eligible. Informed consent was obtained from all subjects. The study adhered to the tenets of the Declaration of Helsinki and was approved by the U.C. Berkeley Committee for the Protection of Human Subjects. The study complied with the relevant CONSORT-AI extension guidelines for clinical studies with an artificial intelligence component.

Participants first completed a battery of questionnaires that detailed their demographics, lifestyle factors, and contact lens history, and included several validated instruments for assessing dry eye symptoms. These were the Ocular Surface Disease Index (OSDI), the Standard Patient Evaluation of Eye Dryness (SPEED), the Dry Eye Flow Chart (DEFC), and visual analog scale (VAS) 0–100 ratings of average and end of day dryness and discomfort and their frequencies. Participants then underwent biomicroscopic examinations of the ocular surface and adnexa with white light. Non-invasive tear breakup times were measured with the Medmont E300 corneal topographer (Medmont International PTY LTD, Nunawading, Australia). The OCULUS Keratograph was used to measure tear meniscus height and for grading of bulbar and limbal hyperemia. The LipiView interferometer (TearScience, Morrisville, NC, USA) was used to measure tear lipid layer thickness. These non-invasive clinical tests were followed by the instillation of 1 µl of 1% fluorescein solution for assessing fluorescein tear breakup times. A biomicroscopic assessment of ocular surface staining was made after instillation of fluorescein and Lissamine Green dyes (10 µl of ~ 1%). Meibomian glands were expressed and meibography images were then captured with the OCULUS Keratograph 5 M (OCULUS, Arlington, WA).

**Fig. 1:**
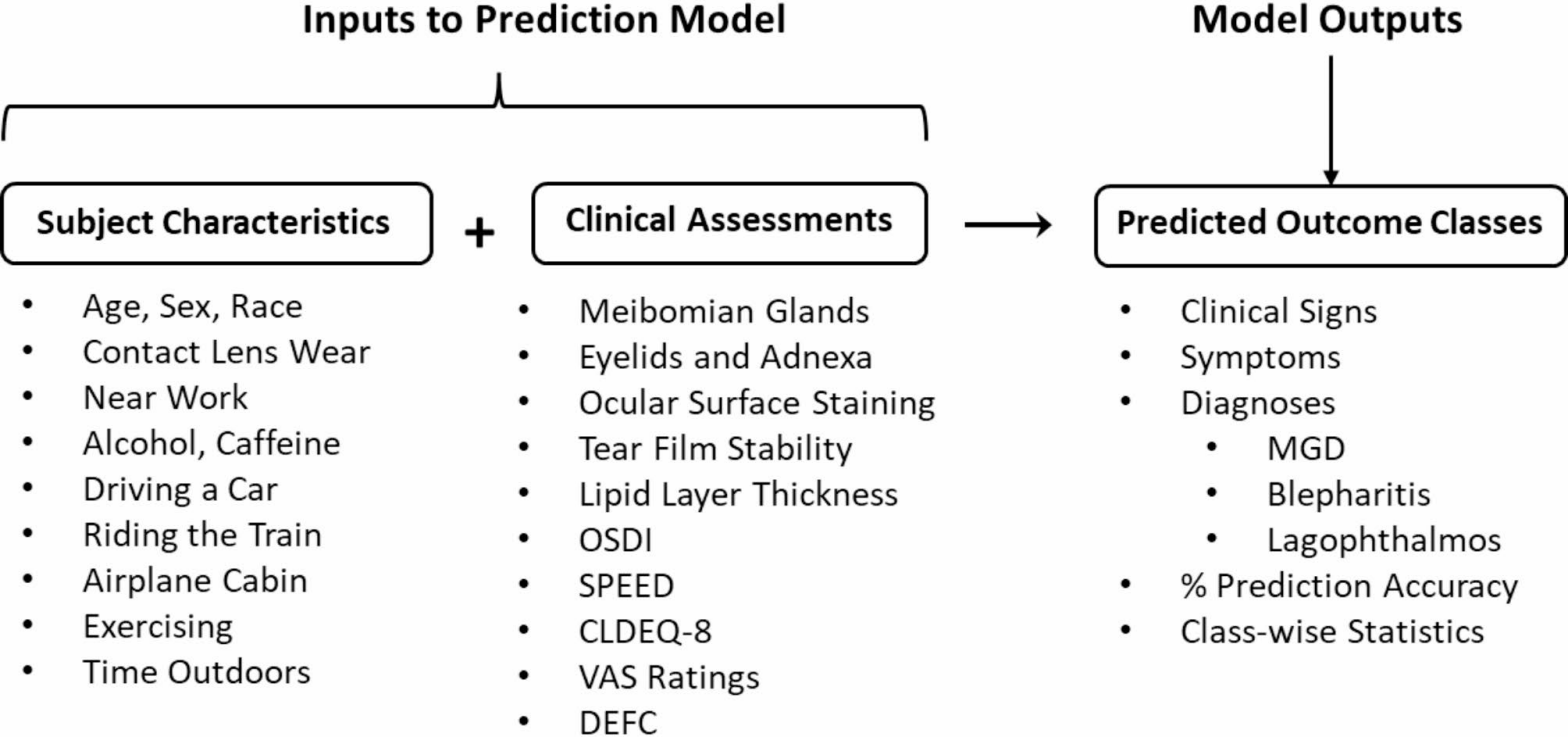
Inputs and outputs for the DE-related outcome prediction models. MGD, Meibomian gland dysfunction; OSDI, Ocular surface disease index; SPEED, Standard patient evaluation of eye dryness; CLDEQ-8, 8-item contact lens dry eye questionnaire; VAS, Visual analog scale; DEFC, Berkeley dry eye flow chart.

The machine learning methodology employed in this study is reported in detail elsewhere^6^. Briefly, a machine learning model was developed to segment Meibomian gland morphological features from meibography images and combine them with subject characteristics, clinical assessments, and symptom scores as inputs to a prediction model. The prediction model then performs classifications into DE-related outcome categories using logistic regression. A depiction of the input features (i.e., the subject, clinical, and symptom variables available as potential predictors) and the output features (i.e., the predicted DE-related outcome classes) is provided in Fig. 1. Some outcomes have natural predicted classes, such as a diagnosis of blepharitis (Yes/No) or eyelid notching (Present/Absent). The predicted classes for continuous and ordinal outcomes were defined based on published thresholds where available^9–14^, and on clinical expertise and standard practice where not. Details of all clinical assessments, symptomatology instruments, and clinician diagnoses are provided in Appendix 1.

**Fig. 2:**
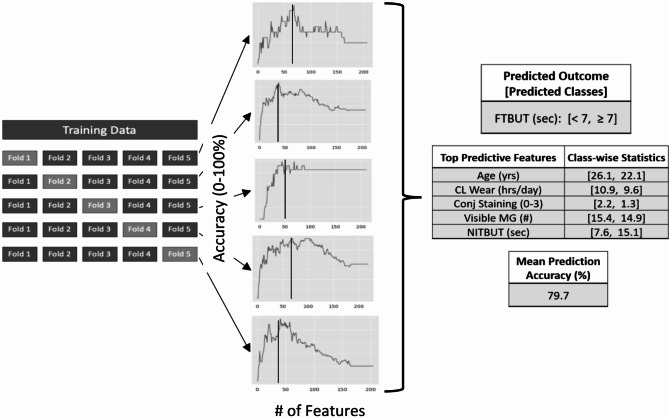
Training process for the DE-related outcome prediction models. FTBUT, Fluorescein tear breakup time; NITBUT, Non-invasive tear breakup time; Conj, Conjunctival; MG, Meibomian glands.

To train the prediction models for each DE-related outcome, data were divided into 5 randomly selected folds, with 4 folds used to train the model and the 5 th used for validation. The models were first trained using all available variables as potential predictive features, then the least weighted feature (i.e., the variable with the lowest coefficient value) was pruned and the model retrained on the remaining features. This process was repeated until only a single predictor remained. From that set of trained models, the one with the highest cross-validation accuracy was selected. To further improve the generalizability of the modeling results, the entire training-pruning-retraining process was repeated using each of the original 5 folds as the validation set. The coefficient values for the 5 best-accuracy models were then aggregated and ranked to determine the most heavily weighted features used for predicting each DE-related outcome. This makes it less likely for the model outputs to be entirely dependent on the makeup of a single validation set. Finally, the class-wise mean values of the predictors stratified on outcome classes were reported, along with the mean cross-validation accuracy. The overall process and an example of the model output are shown in Fig. 2.

## Results

### Subjects

This study utilized 726 clinical records from 363 subjects. The mean (SD) age was 26.6 (12.1) yrs with a range of 18 to 71 yrs. Subjects were 67.2% female, 32.8% male; 46.8% contact lens wearers, 53.2% non-wearers; 43.8% of Asian race, 56.2% of non-Asian race. The distinction between Asian and non-Asian races is based on well-established differences in eyelid anatomy^15^, tear film stability^16^, and DE symptoms^17^. The Asian racial group included subjects of Chinese, Japanese, Korean, and Southeast Asian descent. The non-Asian group consisted primarily of Caucasian subjects, with small minorities of African, Hispanic, and mixed-race subjects.

### Demographic characteristics

Greater age was a heavily weighted predictor of several clinical signs, including eyelid notching, Line of Marx (LoM) anterior displacement, and fluorescein tear breakup time (FTBUT; Table 1). The model for eyelid notching achieved 95.9% prediction accuracy with a 19.6 year greater mean age for subjects with notching. The model for anterior displacement of the LoM achieved 86.8% prediction accuracy with a mean 6.0 year greater age among those with moderate to severe LoM displacement. Among Asian subjects, greater age was a heavily weighted predictor of FTBUT < 6.7 s with a model accuracy of 79.7%.

**Table 1:** Clinical signs predicted by machine learning models that identify lifestyle features as heavily weighted predictors.

Predicted outcomes: clinical signs			
Predicted outcome [predicted classes]	Predictive lifestyle features	Class-wise means	Accuracy (%)
Eyelid notching [absent, present]	Age (yrs)	[27.07, 46.73]	95.92
Eyelid margin erythema: UL [< 2, ≥ 2]	Near work (hrs/day)	[7.25, 8.28]	98.65
Meibum quality: UL, central [< 18, ≥ 18]	Near work (hrs/day)	[7.24, 8.22]	96.05
Meibum quality: LL, entire [< 36, ≥ 36]	Alcoholic beverages (#/wk)	[1.66, 0.68]	93.99
LoM: anterior displacement, UL [< 2, ≥ 2]	Age (yrs)	[26.92, 32.88]	86.82
LoM: anterior displacement, LL [< 2, ≥ 2]	Airplane cabin exposure (hrs/mo)	[1.28, 0.55]	83.00
LWE: length [< 2, ≥ 2]	CL wear history (yrs)	[9.91, 10.17]	92.36
LWE: width [< 2, ≥ 2]	Time exercising (hrs/wk)	[4.60, 3.38]	92.86
Lipid layer thickness (nm) [≤ 60, > 60]	CL wear history (yrs)	[10.64, 9.29]	66.87
Corneal staining: extent [< 2, ≥ 2]	Time outdoors (hrs/day)	[2.72, 2.26]	91.24
Non-invasive TBUT (s): Asian [< 9.0, ≥ 9.0]	Near work (hrs/day)	[8.19, 7.05]	80.35
Fluorescein TBUT (s): Asian [< 6.7, ≥ 6.7]	Age (yrs)	[26.05, 22.11]	79.74
	CL wear duration (hrs/day)	[10.91, 9.59]	
Fluorescein TBUT (s): Non-Asian [< 9.2, ≥ 9.2]	CL wear freq (days/wk)	[5.78, 5.29]	87.39
Fluorescein TBUT (s): all subjects [< 10.0, ≥ 10.0]	CL wear freq (days/wk)	[6.03, 5.64]	84.55

UL, Upper lid; LL, Lower lid; LoM, Line of Marx; LWE, Lid wiper epitheliopathy, TBUT, Tear breakup time; CL, Contact lens; Freq, Frequency.

Age was also a heavily weighted predictor of several DE-related symptoms. Ocular dryness severity and frequency rated on visual analog scales (VAS; Table 2) included age as a heavily weighted predictor. Subjects with the worst average dryness severity averaged 6.9 yrs older than those with the least severe dryness. For severity of end-of-day dryness, subjects with the highest severity averaged 6.7 yrs older. Subjects with the most frequent dryness symptoms averaged 8.0 yrs older that those with the least frequent dryness. Frequency of end-of-day dryness was similar with a 7.0 year greater mean age among those with the most frequent dryness. Interestingly, age was a heavily weighted predictor for all VAS ratings of dryness, but not for any VAS ratings of discomfort.

**Table 2:** Subjective symptoms predicted by machine learning models that identify lifestyle features as heavily weighted predictors.

Predicted outcomes: symptoms			
Predicted outcome [predicted classes]	Predictive lifestyle features	Class-wise means	Accuracy (%)
OSDI score [≤ 12, > 12 ≤ 23, > 23]	Car driving exposure (hrs/wk)	[2.07, 5.29, 3.38]	68.09
	CLW comfortable duration (hrs/day)	[9.01, 8.19, 7.80]	
	Train riding exposure (hrs/wk)	[1.24, 0.71, 1.99]	
SPEED II score [≤ 4, > 4]	CLW comfortable duration (hrs/day)	[9.04, 8.27]	74.47
	CLW history (yrs)	[9.85, 10.08]	
	Alcoholic beverages (#/wk)	[0.99, 1.97]	
VAS comfort [< 75, ≥ 75 < 83, ≥ 83]	CLW comfortable duration (hrs/day)	[7.52, 8.78, 9.31]	65.35
VAS discomfort frequency [< 10, ≥ 10 < 17, ≥ 17]	CLW comfortable duration (hrs/day)	[9.24, 8.96, 7.89]	60.71
	Airplane cabin exposure (hrs/mo)	[0.81, 1.70, 1.22]	
	Time exercising (hrs/wk)	[4.80, 3.99, 4.13]	
	Alcoholic beverages (#/wk)	[0.96, 1.81, 1.97]	
VAS EOD comfort [< 59, ≥ 59 < 76, ≥ 76]	CLW comfortable duration (hrs/day)	[8.02, 8.48, 9.03]	63.26
	Alcoholic beverages (#/wk)	[2.01, 2.12, 1.06]	
	Car driving exposure (hrs/wk)	[3.96, 2.65, 2.51]	
VAS EOD discomfort frequency [< 17, ≥ 17 < 32, ≥ 32]	Alcoholic beverages (#/wk)	[1.00, 1.98, 2.05]	63.09
	CLW duration (hrs/day)	[10.39, 10.81, 10.28]	
VAS dryness [< 20, ≥ 20 < 43, ≥ 43]	CLWr comfortable duration (hrs/day)	[9.18, 8.23, 7.67]	66.13
	Age (yrs)	[25.87, 28.01, 32.75]	
	Car driving exposure (hrs/wk)	[2.58, 2.22, 4.60]	
VAS dryness frequency [< 19, ≥ 19 < 48, ≥ 48]	CLW comfortable duration (hrs/day)	[9.14, 8.25, 7.40]	67.24
	Age (yrs)	[26.27, 27.27, 34.27]	
VAS EOD dryness [< 31, ≥ 31 < 61, ≥ 61]	CLW comfortable duration (hrs/day)	[8.98, 7.92, 7.99]	70.29
	Age (yrs)	[26.37, 26.90, 33.11]	
VAS EOD dryness frequency [< 32, ≥ 32 < 65, ≥ 65]	CLW comfortable duration (hrs/day)	[8.82, 8.63, 7.90]	70.18
	Age (yrs)	[26.75, 26.50, 33.72]	
DEFC any dryness: CLW [ASYM, CLIDE, DE]	CLW comfortable duration (hrs/day)	[12.92, 8.77, 8.56]	61.11
	Time exercising (hrs/wk)	[4.31, 3.95, 3.74]	
DEFC debilitating dryness: CLW [ASYM, CLIDE, DE]	CLW comfortable duration (hrs/day)	[11.75, 8.13, 7.60]	63.93
	Alcoholic beverages (#/wk)	[1.09, 1.61, 2.43]	
	Time exercising (hrs/wk)	[3.88, 3.95, 3.95]	
DEFC debilitating dryness: non-CLW [ASYM, DE]	Car driving exposure (hrs/wk)	[2.26, 5.23]	86.54
	Alcoholic beverages (#/wk)	[1.31, 2.27]	
CLDEQ8 score [< 12, ≥ 12]	CLW comfortable duration (hrs/day)	[10.56, 7.89]	76.31
	CLW duration (hrs/day)	[11.05, 10.69]	
	Time outdoors (hrs/day)	[2.66, 2.10]	
	Caffeinated drinks (#/day)	[0.75, 0.93]	

OSDI, Ocular surface disease index; SPEED, Standard patient evaluation of eye dryness; VAS, Visual analog scale; EOD, End of day; DEFC, Dry eye flow chart; CLW, Contact lens wear; ASYM, Asymptomatic; CLIDE, Contact lens-induced dry eye; DE, Dry eye; CLDEQ8, 8-Item contact lens dry eye questionnaire.

The prediction model for a diagnosis of blepharitis included age as heavily weighted feature (Table 3), and achieved 73.7% prediction accuracy. Subjects with blepharitis averaged approximately 5.4 yrs older than those without blepharitis.

**Table 3:** Clinician diagnoses predicted by machine learning models that identify lifestyle features as heavily weighted predictors.

Predicted outcomes: diagnoses			
Predicted outcome [predicted classes]	Predictive lifestyle features	Class-wise means	Accuracy (%)
Meibomian gland dysfunction [Yes, No]	CL wear history (yrs)	[9.85, 10.10]	74.38
Blepharitis [Yes, No]	Age (yrs)	[30.36, 24.95]	73.67
Lagophthalmos [Yes, No]	Airplane cabin exposure (hrs/mo)	[1.64, 0.90]	80.07

CL, Contact lens.

Sex and race were not heavily weighted features in any prediction models of signs, symptoms, or diagnoses.

### Contact lens wear

Contact lens wear (CLW) patterns were heavily weighted in several prediction models. Some measures of CLW, specifically history (yrs) and frequency (days/wk), although heavily weighted in some models, revealed only minimal differences between subjects with and without signs or symptoms (e.g., a mean of 0.25 yrs longer CLW among those with MGD).

Longer CLW duration (hrs/day) was a heavily weighted predictor of FTBUT among Asian subjects (79.7% accuracy) with approximately 1.3 h/day longer wear for subjects with shorter FTBUT. Although the difference appears minimal, it should kept in mind that it is equivalent to 9.1 h/wk less CLW among those with better tear film stability. CLW duration was not a heavily weighted feature in any symptom or diagnosis predictions.

In contrast, the duration of *comfortable* CLW (hrs/day) was an important predictor for every subjective measure of symptoms studied. For Ocular Surface Disease Index (OSDI) score, comfortable CLW averaged 1.2 h/day longer among those with the mildest symptoms. Longer comfortable wearing time was predictive of lower VAS ratings of ocular discomfort and dryness severity and frequency, both overall and at end-of-day. Subjects who were classified as asymptomatic for DE with the Berkeley Dry Eye Flow Chart (DEFC) averaged 12.9 comfortable hrs/day of lens wear, contact lens-induced DE subjects averaged 8.8 h/day, and subjects with physiological DE averaged 8.6 h/day. Comfortable CLW duration was also a heavily weighted predictor of DEFC debilitating symptoms in the highest accuracy model of any symptom assessment (86.5%). Asymptomatic subjects averaged 11.8 h/day of comfortable lens wear, subjects with debilitating contact lens-induced DE averaged 8.1 h/day, and subjects with debilitating physiological DE averaged 7.6 h/day. Finally, Contact Lens Dry Eye Questionnaire (CLDEQ-8) score was predicted with 76.3% accuracy with a comfortable contact lens wearing time of 2.7 h/day longer among subjects with no or mild symptoms.

### Detrimental lifestyle behaviors

There are a number of lifestyle behaviors that are known or generally considered to have positive or negative effects on health that may also have effects on the ocular surface and/or subjective symptoms. A greater amount of near work (hrs/day) was found to be a heavily weighted predictor of eyelid margin erythema in a model achieving 98.7% prediction accuracy. Among Asian subjects, those with non-invasive tear breakup time (NITBUT) < 9.0 s averaged 8.2 h of near work per day and those with breakup times ≥ 9.0 s averaged 7.1 h (80.4% accuracy).

Consuming alcoholic beverages was a heavily weighted predictor of meibum quality, averaging 1.0 drinks more per week among those with poor meibum quality (94.0% accuracy). Alcoholic beverage consumption was a heavily weighted feature in several symptom prediction models. Subjects with high Standard Patient Evaluation of Eye Dryness (SPEED II) scores (worse symptoms) averaged 1.0 drinks per week more than those with mild or no symptoms (74.5% accuracy). The number of alcoholic drinks per week was also a heavily weighted predictor of VAS ratings of ocular discomfort frequency, end-of-day discomfort, and frequency of end-of-day discomfort. In each of those models, subjects with severe and frequent symptoms consumed approximately 1.0 drinks per week more on average. The model of DEFC debilitating symptoms among contact lens wearers showed that asymptomatic lens wearers averaged 1.1 alcoholic drinks per week, those with contact lens-induced DE 1.6 drinks per week, and those with physiological DE 2.4 drinks per week.

### Beneficial lifestyle behaviors

Time exercising (hrs/wk) was a heavily weighted predictor of lid wiper epitheliopathy (LWE; 92.9% accuracy), averaging 1.2 h/wk more exercise among subjects with no or mild LWE. In terms of symptoms, subjects with the most frequent VAS discomfort exercised approximately 0.7 h/wk less, and subjects classified as symptomatic by the DEFC exercised approximately 0.6 h/wk less.

Less time spent outdoors (hrs/day) was a heavily weighted predictor of corneal staining extent (91.2% accuracy), and of CLDEQ-8 score (76.3% accuracy). Subjects with moderate to severe corneal staining extent averaged 0.5 fewer hours per day outdoors. Contact lens wearers with high CLDEQ-8 scores (worse symptoms) spent approximately 0.6 fewer hours per day outdoors.

### Environmental exposures

More exposure to airplane cabin environments (hrs/mo) was a heavily weighted predictor for anterior displacement of the LoM (83.0% accuracy) and a diagnosis of lagophthalmos (80.1% accuracy). More airplane cabin exposure was also a heavily weighted predictor of more frequent ocular discomfort in VAS ratings. The mean differences in airplane cabin exposure between those with and without signs or symptoms were minimal at approximately 0.7 h/mo in all models.

More time riding the train (hrs/wk) was predictive of a higher OSDI score, and subjects with the highest OSDI scores (worse symptoms) were exposed to riding the train approximately 0.8 h/wk more than those with the lowest OSDI scores. Driving a car (hrs/wk) was predictive of several assessments of subjective symptoms. Subjects with the highest OSDI scores averaged approximately 1.3 h/wk more driving time. For VAS severity of end-of-day ocular discomfort, subjects with the lowest comfort ratings drove a car on average 1.5 h/wk more. Subjects with the highest VAS dryness severity ratings averaged approximately 2.0 h/wk more driving time. Among non-contact lens wearers, subjects symptomatic for debilitating DE by DEFC classification averaged approximately 3 h/wk more exposure to driving a car than did asymptomatic subjects (86.5% accuracy).

## Discussion

In this study, machine learning models were trained to take subject characteristics, lifestyle behaviors and risk exposures, clinical assessments of the ocular surface, tear film and eyelids, and symptom scores from validated DE instruments, and combine them in prediction models of DE-related outcomes. Lifestyle factors were found to be among the most heavily weighted features used by the models to predict a number of clinical signs, subjective symptoms, and diagnoses related ocular surface disease. Prediction accuracies for DE-related symptoms ranged from 60.7 to 86.5%, for diagnoses from 73.7 to 80.1%, and for clinical signs from 66.9 to 98.7%.

Greater age was a heavily weighted predictor for clinical signs including the presence of eyelid notching, anterior displacement of the LoM, and shorter FTBUT among Asian subjects. Greater age was also a heavily weighted predictor for VAS dryness severity and frequency ratings, both throughout the day and at end-of-day, as well as for a clinical diagnosis of blepharitis. There is evidence to suggest that the LoM can shift due to aging, and due to the presence of DE^14,18^. Eyelid margin irregularities such as notching are frequently observed in cases of blepharitis and MGD^19,20^, both conditions known to be related to aging^21–24^. It has been well documented that symptoms of DE and MGD are on average more severe, frequent, and prevalent among older populations^22,25–27^.

More years of CLW was a heavily weighted predictor in models of LWE, a thinner lipid layer, a higher SPEED II score, and a diagnosis of MGD, all of which are in agreement with the literature^28–32^. In general, however, the interclass differences in these models were very small (0.2–1.4 yrs of CLW). Similarly, CLW frequency (days/wk) was a heavily weighted predictor of unstable vs. stable FTBUT^33^ but with small interclass differences (0.4–0.5 days/wk). These results illustrate how very small differences that are not considered to be of importance to clinicians can still be heavily weighted features in machine learning predictions^7^.

Duration of CLW (hrs/day) was a heavily weighted feature in predicting FTBUT among Asian subjects. In contrast, while the duration of *comfortable* CLW (hrs/day) was not a heavily weighted predictor for any clinical signs, it was an important predictor for every subjective measure of symptoms studied^34^. Asymptomatic subjects averaged 0.8–4.4 more hrs/day of comfortable CLW. Total hrs/day of CLW is not always informative because corneal desensitization, wearer commitment, lifestyle needs, and individual pain sensitivity level can result in continuing wear far beyond the onset of symptoms. Hrs/day of comfortable CLW was a far better predictor of symptoms. Clinicians should ask symptomatic contact lens patients about their comfortable wearing time and distinguish it from their total wearing time^35^.

It is important to point out that with these machine learning prediction models the direction of causality is generally unknown, but sometimes can be inferred logically. For example, there was longer CLW duration (hrs/day) among Asian subjects with shorter FTBUT. Other than by chance (e.g., some unknown sampling bias) or the action of unknown latent variables, there is no reason to think that worse tear film stability would cause contact lens wearers to wear their lenses longer. The fact that those with shorter FTBUT were actually wearing their lenses longer implies that the direction of causation is more likely from longer CLW to shorter FTBUT and not the reverse.

Amount of near work (hrs/day) was a heavily weighted predictor of eyelid margin erythema among all subjects and shorter NITBUT among Asian subjects. Subjects with erythema or reduced tear film stability averaged slightly over an hour per day more near work. Frequent near work is a well-known risk factor for DE, particularly in the context of digital display use^36–38^. While there is little information on the effects of near work on the eyelids, Wu et al. found that an eyelid margin abnormality score was positively correlated with time using a visual display terminal, and that FTBUT, corneal staining, and OSDI score were all significantly worse in a cohort using visual display terminals for more than 4 h per day^39^. Most studies of near work and tear film stability have employed FTBUT as the outcome measure. Khezrzade et al., however, did find that NITBUT was significantly reduced after 30 min of reading^40^. To our knowledge, the machine learning results presented here represent the only other evidence of the effects of sustained near work on non-invasive measurements of tear film stability, and that sustained near work may ultimately have effects on the eyelid margin.

Consuming caffeinated beverages was a heavily weighted predictor only for CLDEQ-8 score, and only with an average of 0.2 drinks per day more among those with a higher score. Caffeinated beverage consumption was not predictive of any other signs, symptoms, or diagnoses. Most studies have found either no relationship between caffeine consumption and DE^41^, or a possible protective effect^1,42,43^. Consumption of alcohol on the other hand was a heavily weighted predictor of poor meibum quality and of worse DE symptoms on several questionnaire instruments. Subjects with poor meibum quality averaged 1.0 drink more per week, and symptomatic subjects averaged 1.0–1.3 drinks more per week. Although the effect size appears to be small, it should be kept in mind that it is equivalent to 52–68 drinks more over the course of a year. The literature on the effects of alcohol on the signs and symptoms of DE is largely equivocal^1^. Some studies have found alcohol consumption to be linked to tear film deterioration, reduced tear volume, increased osmolarity, and worse DE symptoms^43,44^. Other studies have found alcohol to be a non-factor in DE^42,45,46^, and a few studies have reported a protective effect against DE^41,47^. To our knowledge this is the first study to link alcohol consumption to lower quality meibum. Magno et al. found that alcohol consumption significantly increased the risk of DE in women but not in men, possibly due to differences the hormone androgen, the deficiency of which has been linked to MGD^44^. In men, it has been shown that excessive or chronic alcohol consumption can reduce serum testosterone^48^. Modeling the interaction of alcohol consumption and sex was not performed in this study and may deserve further investigation.

More time exercising was found to be a heavily weighted predictor of less LWE. LWE is associated with sub-clinical inflammation^49^, and exercise has been linked to reduced tear concentrations of several cytokines and other markers of inflammation or oxidative stress^50–52^. Aerobic exercise has been shown to promote tear secretion and improves tear film stability in dry eye patients^50,53^, and tear film instability has been linked to LWE^28^. Other studies have also demonstrated a link between a lack of exercise (i.e., sedentary lifestyle) and risk of DE. Sedentary behavior has been associated with reduced tear breakup time, lower tear volume, and risk of DE^50–53^. It has been speculated that exercise increases parasympathetic stimulation of the lacrimal gland and acinar blood vessels, increasing secretion of electrolytes and aqueous^1^.

Approximately 2.5 h more per week spent outdoors was found to be a heavily weighted predictor of lesser corneal staining extent, and of lower CLDEQ-8 score among contact lens wearers. Some studies have found time outdoors to be a risk factor for DE^46,54^, often related to extreme heat or cold conditions^38^ or excessive wind^55^. Other studies have found time spent outdoors to be a non-factor in risk for DE^45^. Rodriguez et al. found that time spent on indoor work was associated with a decreased blink rate^56^, which is well known to be an etiological factor in DE. In this study, a post-hoc analysis showed that our subjects who spent more time outdoors were also doing less near work on average (thus presumably blinking more), and exercising significantly more.

More time riding the train was a heavily weighted predictor of higher OSDI score. More time driving a car was a heavily weighted predictor of higher symptom scores including OSDI score, VAS ratings, and DEFC classification. Symptomatic subjects averaged 0.8–3.0 more hours per week exposure. There are likely similarities and differences in the mechanisms of DE symptoms in these two types of exposure. While there are studies on how DE affects the ability to drive^26^, there are relatively few studies of car driving or train riding as a causative or risk factor for DE. Guillon et al. found a greater incidence of symptoms among DE subjects after riding the subway and after driving a car for both contact lens wearers and non-wearers^57^. Rodriguez et al. found increased levels of ocular discomfort and a reduced interblink period associated with driving a car^56^. The link between DE and these exposures could be due to the inside environment (e.g., windows open or closed; heater or air conditioner settings; fan settings; environmental contaminants or cleaning product irritants), which could apply to both cars and trains. It could also be due to extended visual tasking while driving for extended periods which reduces the interblink period^56^, while extended visual tasking at distance would likely not apply to riding the train.

As with any study there are some limitations and caveats. Larger datasets for some sparse variables are likely to improve prediction accuracy further, especially for symptoms. There are numerous other likely important lifestyle behaviors and exposures that were not addressed in this study, including obesity, dietary habits, health and wellness supplements, sleep patterns, and a wide variety of ocular and systemic medications, to name a few. With respect to interpreting the output of these machine learning models, it is important to understand that the features in the highest accuracy prediction models and their weights relative to other variables are determined by the model without human intervention. These are not to be confused with classical statistical models in which coefficients are determined for a sample of data to estimate population parameters under a set of assumptions. The heavily weighted features presented in this work are not to be interpreted as independent factors, and confounders and interactions were not modeled statistically. These exploratory models are data-driven and the convolutional neural network enables the model to determine both the optimal set of variables and their relative weights in order to make the highest accuracy predictions. The makeup of our study population should also be kept in mind when interpreting the results. Participants were mostly young, healthy members of the university campus and surrounding community. Dry eye and other ocular surface diseases are more prevalent in older populations, and the models may not be generalizable to these populations without additional training data from a larger number of older participants. It should also be kept in mind that the data on lifestyle factors are self-reported and thus subject to faulty memory, or under- or over-estimation due to perceived social response (e.g., many people will underestimate their alcohol consumption even on anonymized questionnaires). Future work would benefit from modeling interactions among demographic and risk factors to determine if predictive relationships are the same for different ages, sexes, and races.

## Conclusions

In this study a novel machine learning approach was employed to predict DE-related outcomes using combined clinical, symptom, and lifestyle data. The algorithm relied heavily on a number of subject characteristic, lifestyle behavior, and environmental exposure variables to make the highest accuracy predictions. Age was a heavily weighted feature in predictions of eyelid notching, LoM anterior displacement, and FTBUT, as well as VAS symptom ratings and a clinician diagnosis of blepharitis. Contact lens wear patterns were heavily weighted features in predictions of FTBUT and subjective ratings of DE symptoms. Some generally beneficial or detrimental behaviors were shown to also be important predictors of ocular signs and symptoms, including time spent in near work, alcohol consumption, exercise, and time spent outdoors. Exposure to riding the train and driving a car were predictors of DE-related symptoms but not clinical signs. These results illustrate the importance of lifestyle, subject, and environmental characteristics in ocular surface health and disease, and underscore the emerging consensus that the impact of these factors in clinical care and clinical research must be taken into account with greater rigor than has largely been the case to date.

## Electronic supplementary material

Below is the link to the electronic supplementary material.

Supplementary Material 1

## Data Availability

De-identified data will be made available upon request for research purposes only with valid Data Transfer and Use Agreements (DTUA) required for sharing protected human subject data. Contact the Corresponding Author (MCL) for data inquiries.

## References

[1] Stapleton, F. et al. TFOs lifestyle: Impact of societal challenges on the ocular surface. *Ocul Surf.* **28**, 165–199 (2023). 37062429 10.1016/j.jtos.2023.04.006 PMC10102706

[2] Tan, T. F. et al. Artificial intelligence and digital health in global eye health: Opportunities and challenges. *Lancet Glob. Health* **11**(9), E1432–E1443 (2023). 37591589 10.1016/S2214-109X(23)00323-6

[3] Fineide, F., Arita, R. & Utheim, T. P. The role of meibography in ocular surface diagnostics: A review. *Ocul Surf.* **19**, 133–144 (2021). 32416235 10.1016/j.jtos.2020.05.004

[4] Wang, J. et al. Quantifying Meibomian gland morphology using artificial intelligence. **98**(9), 1094–1103 (2021). 10.1097/OPX.0000000000001767 PMC8484036 34469930

[5] Wang, J., Yeh, T. N., Chakraborty, R., Yu, S. X. & Lin, M. C. A deep learning approach for Meibomian gland atrophy evaluation in meibography images. *Transl. Vis. Sci. Technol.* **8**(6), 37 (2019). 31867138 10.1167/tvst.8.6.37 PMC6922272

[6] Graham, A. D. et al. A machine learning approach to predicting dry eye-related signs, symptoms and diagnoses from meibography images. *Heliyon* **10**(17), e36021 (2024). 39286076 10.1016/j.heliyon.2024.e36021 PMC11403426

[7] Fineide, F. A., Storås, A. M., Riegler, M. A. & Utheim, T. P. Predicting Meibomian gland dropout and feature importance analysis in explainable artificial intelligence. In *2023 IEEE 36th International Symposium on Computer-Based Medical Systems (CBMS)* 366–373 (2023).

[8] Yeh, C.-H., Yu, S. X. & Lin, M. C. Meibography phenotyping and classification from unsupervised discriminative feature learning. *Transl. Vis. Sci. Technol.* **10**(2), 4 (2021). 34003889 10.1167/tvst.10.2.4 PMC7873493

[9] Cochener, B., Cassan, A. & Omiel, L. Prevalence of Meibomian gland dysfunction at the time of cataract surgery. *J. Cataract. Refract. Surg.* **44**(2), 144–148 (2018). 29587971 10.1016/j.jcrs.2017.10.050

[10] Isreb, M. A. et al. Correlation of lipid layer thickness measurements with fluorescein tear film breakup time and Schirmer’s test. *Eye* **17**(1), 79–83 (2003). 12579175 10.1038/sj.eye.6700224

[11] Graham, A. D., Lundgrin, E. L. & Lin, M. C. The Berkeley dry eye flow chart: A fast, functional screening instrument for contact lens-induced dryness. *PLoS One* **13**(1), e0190752 (2018). 29364947 10.1371/journal.pone.0190752 PMC5783349

[12] Asiedu, K. et al. Ocular surface disease index (OSDI) versus the standard patient evaluation of eye dryness (SPEED): A study of a nonclinical sample. *Cornea* **35**(2), 175–180 (2016). 26655485 10.1097/ICO.0000000000000712

[13] Chalmers, R. L., Keay, L., Hickson-Curran, S. B. & Gleason, W. J. Cutoff score and responsiveness of the 8-item contact lens dry eye questionnaire (CLDEQ-8) in a large daily disposable contact lens registry. *Contact Lens Anterior Eye* **39**(5), 342–352 (2016). 27131891 10.1016/j.clae.2016.04.005

[14] Lievens, C. W., Norgett, Y., Briggs, N., Allen, P. M. & Vianya-Estopa, M. Impact of improper approach to identify lid wiper epitheliopathy (LWE). *Clin. Ophthalmol.* **14**, 3039–3047 (2020). 33116355 10.2147/OPTH.S273524 PMC7547802

[15] Jeong, S., Lemke, B. N., Dortzbach, R. K., Park, Y. G. & Kang, H. K. The Asian upper eyelid: An anatomical study with comparison to the Caucasian eyelid. *Arch. Ophthalmol.* **117**(7), 907–912 (1999). 10408455 10.1001/archopht.117.7.907

[16] Wang, M. T. M. & Craig, J. P. Natural history of dry eye disease: Perspectives from inter-ethnic comparison studies. *Ocul. Surf.* **17**(3), 424–433 (2019). 30965124 10.1016/j.jtos.2019.03.004

[17] Tran, N., Graham, A. D. & Lin, M. C. Ethnic differences in dry eye symptoms: Effects of corneal staining and length of contact lens wear. *Contact Lens Anterior Eye* **36**, 281–288 (2013). 23850062 10.1016/j.clae.2013.06.001

[18] Yamaguchi, M. et al. Marx line: Fluorescein staining line on the inner lid as indicator of Meibomian gland function. *Am. J. Ophthalmol.* **141**(4), 669-669.e8 (2006). 16564801 10.1016/j.ajo.2005.11.004

[19] Ha, M. et al. Relationship between eyelid margin irregularity and Meibomian gland dropout. *Ocul. Surf.* **19**, 31–37 (2021). 33246034 10.1016/j.jtos.2020.11.007

[20] Gurnani, B. & Kaur, K. *Meibomian Gland Disease* StatPearls (StatPearls Publishing, 2023). 35593799

[21] Abelson MB, Ousler G, Shapiro A, Rimmer D. The form and function of Meibomian glands. *Rev Ophthalmol.* (2016).

[22] Arita, R. et al. Meibomian gland dysfunction and dry eye are similar but different based on a population-based study: The Hirado-Takushima study in Japan. *Am. J. Ophthalmol.* **207**, 410–418 (2019). 30851269 10.1016/j.ajo.2019.02.024

[23] Nemet, A. Y., Vinker, S. & Kaiserman, I. Associated morbidity of blepharitis. *Ophthalmology* **118**(6), 1062–1068 (2011). 21276617 10.1016/j.ophtha.2010.10.015

[24] Sędzikowska, A., Osęka, M. & Skopiński, P. The impact of age, sex, blepharitis, rosacea, and rheumatoid arthritis on Demodex mite infection. *Arch. Med. Sci.* **2**, 353–356 (2018). 10.5114/aoms.2016.60663 PMC5868666 29593809

[25] Titiyal, J. S., Falera, R. C., Kaur, M., Sharma, V. & Sharma, N. Prevalence and risk factors of dry eye disease in North India: Ocular surface disease index-based cross-sectional hospital study. *Indian J. Ophthalmol.* **66**(2), 207–211 (2018). 29380759 10.4103/ijo.IJO_698_17 PMC5819096

[26] Stapleton, F. et al. TFOS DEWS II epidemiology report. *Ocul. Surf.* **15**(3), 334–365 (2017). 28736337 10.1016/j.jtos.2017.05.003

[27] Yeh, T. N., Graham, A. D. & Lin, M. C. Relationships among tear film stability, osmolarity, and dryness symptoms. *Optom. Vis. Sci.* **92**(9), e264–e272 (2015). 26154693 10.1097/OPX.0000000000000649 PMC4924532

[28] Li W, Yeh TN, Leung T, Yuen T, Lerma M, Lin MC. The relationship of lid wiper epitheliopathy to ocular surface signs and symptoms. 10.1167/iovs.17-23639 29677348

[29] Mann, A. & Tighe, B. Contact lens interactions with the tear film. *Exp. Eye Res.* **117**, 88–98 (2013). 23886658 10.1016/j.exer.2013.07.013

[30] Rohit, A., Willcox, M. & Stapleton, F. Tear lipid layer and contact lens comfort: A review. *Eye Contact Lens.* **39**(3), 247–253 (2013). 23584045 10.1097/ICL.0b013e31828af164

[31] Molina, K. et al. Not all dry eye in contact lens wear is contact lens-induced. *Eye Contact Lens.* **46**(4), 214–222 (2020). 31517736 10.1097/ICL.0000000000000661

[32] Alghamdi, W. M., Markoulli, M., Holden, B. A. & Papas, E. B. Impact of duration of contact lens wear on the structure and function of the Meibomian glands. *Ophthalmic Physiol. Opt.* **36**(2), 120–131 (2016). 26890701 10.1111/opo.12278

[33] Yeh, T. N. & Lin, M. C. Risk factors for severe Meibomian gland atrophy in a young adult population: A cross-sectional study. *PLoS One* **12**(9), e0185603 (2017). 28957399 10.1371/journal.pone.0185603 PMC5619790

[34] Young, G. et al. Soft contact lens-related dryness with and without clinical signs. *Optom. Vis. Sci.* **89**(8), 1125–1132 (2012). 22820475 10.1097/OPX.0b013e3182640af8

[35] Riley, C., Young, G. & Chalmers, R. Prevalence of ocular surface symptoms, signs, and uncomfortable hours of wear in contact lens wearers: The effect of refitting with daily-wear silicone hydrogel lenses (Senofilcon A). *Eye Contact Lens.* **32**(6), 281–286 (2006). 17099389 10.1097/01.icl.0000224522.04723.7a

[36] Wang, M. T., Muntz, A., Mamidi, B., Wolffsohn, J. S. & Craig, J. P. Modifiable lifestyle risk factors for dry eye disease. *Contact Lens Anterior Eye* **44**(6), 101409 (2021). 33485806 10.1016/j.clae.2021.01.004

[37] Wolffsohn, J. S. et al. Demographic and lifestyle risk factors of dry eye disease subtypes: A cross-sectional study. *Ocul. Surf.* **21**, 58–63 (2021). 33965652 10.1016/j.jtos.2021.05.001

[38] Gayton, J. L. Etiology, prevalence, and treatment of dry eye disease. *Clin. Ophthalmol.* **3**, 405–412 (2009). 19688028 10.2147/opth.s5555 PMC2720680

[39] Wu, H. et al. Meibomian gland dysfunction determines the severity of the dry eye conditions in visual display terminal workers. *PLoS One* **9**(8), e105575 (2014). 25144638 10.1371/journal.pone.0105575 PMC4140788

[40] Khezrzade, S., Ehsaei, A., Momeni-Moghaddam, H., Wollfsoh, J. S. & Abadi, S. O. A. After-effect on tear film quality and quantity of reading on laptop computer screen versus hardcopy. *Clin. Exp. Optom.* (2023). 10.1080/08164622.2023.2241053 37797942

[41] Moss, S. E., Klein, R. & Klein, B. E. K. Long-term incidence of dry eye in an older population. *Optom. Vis. Sci.* **85**(8), 668–674 (2008). 18677233 10.1097/OPX.0b013e318181a947

[42] García-Marqués, J. V., Talens-Estarelles, C., García-Lázaro, S., Wolffsohn, J. S. & Cerviño, A. Systemic, environmental and lifestyle risk factors for dry eye disease in a Mediterranean Caucasian population. *Contact Lens Anterior Eye* **45**, 101539 (2022). 34789408 10.1016/j.clae.2021.101539

[43] Galor, A. et al. TFOS lifestyle: Impact of lifestyle challenges on the ocular surface. *Ocul. Surf.* **28**, 262–303 (2023). 37054911 10.1016/j.jtos.2023.04.008

[44] Magno, M. S. et al. The relationship between alcohol consumption and dry eye. *Ocul. Surf.* **21**, 87–95 (2021). 34029755 10.1016/j.jtos.2021.05.005

[45] Moss, S. E., Klein, R. & Klein, B. E. K. Prevalence and risk factors for dry eye syndrome. *Arch. Ophthalmol.* **118**(9), 1264–1268 (2000). 10980773 10.1001/archopht.118.9.1264

[46] Vidal-Rohr, M., Craig, J. P., Davies, L. N. & Wolffsohn, J. S. The epidemiology of dry eye disease in the UK: The Aston dry eye study. *Contact Lens Anterior Eye* **46**(3), 101837 (2023). 37003925 10.1016/j.clae.2023.101837

[47] Viso, E., Rodriguez-Ares, M. T., Abelenda, D., Oubiña, B. & Gude, F. Prevalence of symptomatic and symptomatic Meibomian gland dysfunction in the general population of Spain. *Invest. Ophthalmol. Vis. Sci.* **53**(6), 2601–2606 (2012). 22427596 10.1167/iovs.11-9228

[48] Smith, S. J., Lopresti, A. L. & Fairchild, T. J. The effects of alcohol on testosterone synthesis in men: A review. *Expert Rev. Endocrinol. Metab.* **18**(2), 155–166 (2023). 36880700 10.1080/17446651.2023.2184797

[49] Efron, N., Brennan, N. A., Morgan, P. B. & Wilson, T. Lid wiper epitheliopathy. *Prog. Retinal Eye Res.* **53**, 140–174 (2016). 10.1016/j.preteyeres.2016.04.004 27094372

[50] Navarro-Lopez, S., Moya-Ramón, M., Gallar, J., Carracedo, G. & Aracil-Marco, A. Effects of physical activity/exercise on tear film characteristics and dry eye associated symptoms: A literature review. *Contact Lens Anterior Eye* **46**(4), 101854 (2023). 37173175 10.1016/j.clae.2023.101854

[51] Kawashima, M. et al. The association between dry eye disease and physical activity as well as sedentary behavior: Results from the Osaka study. *J. Ophthalmol.* **943786**, 1–6 (2014). 10.1155/2014/943786 PMC4248401 25485144

[52] Kojima, T., Dogru, M., Kawashima, M., Nakamura, S. & Tsubota, K. Advances in the diagnosis and treatment of dry eye. *Prog. Retinal Eye Res.* **78**, 100842 (2020). 10.1016/j.preteyeres.2020.100842 32004729

[53] Sun, C. et al. Effects of aerobic exercise on tear secretion and tear film stability in dry eye patients. *BMC Ophthalmol.* **22**(1), 9 (2022). 34983454 10.1186/s12886-021-02230-9 PMC8725542

[54] Kim, Y. et al. Short-term effects of ground-level ozone in patients with dry eye disease: A prospective clinical study. *Cornea* **38**(12), 1483–1488 (2019). 31299662 10.1097/ICO.0000000000002045

[55] Li, J. et al. Prevalence and risk factors of dry eye disease among a hospital-based population in southeast China. *Eye Contact Lens.* **41**(1), 44–50 (2015). 25232992 10.1097/ICL.0000000000000064

[56] Rodriguez, J. D. et al. Blink: Characteristics, controls, and relation to dry eyes. *Curr Eye Res.* **43**(1), 52–66 (2018). 29043838 10.1080/02713683.2017.1381270

[57] Guillon, M. & Maissa, C. Dry eye symptomatology of soft contact lens wearers and nonwearers. *Opt. Vis. Sci.* **82**(9), 829–834 (2005). 10.1097/01.opx.0000178060.45925.5d 16189493

