# Supplementary material for: Artificial intelligence models utilize lifestyle factors to predict dry eye related outcomes
Source: Sci Rep. 2025 Apr 18;15:13378. doi: 10.1038/s41598-025-96778-x (PMC12008223; doi:10.1038/s41598-025-96778-x)
Supplement: Supplementary file 1 — Supplementary Material 1 [file 41598_2025_96778_MOESM1_ESM.docx]

**APPENDIX 1 – Clinical Assessments, Symptom Instruments, Diagnostic Criteria.**

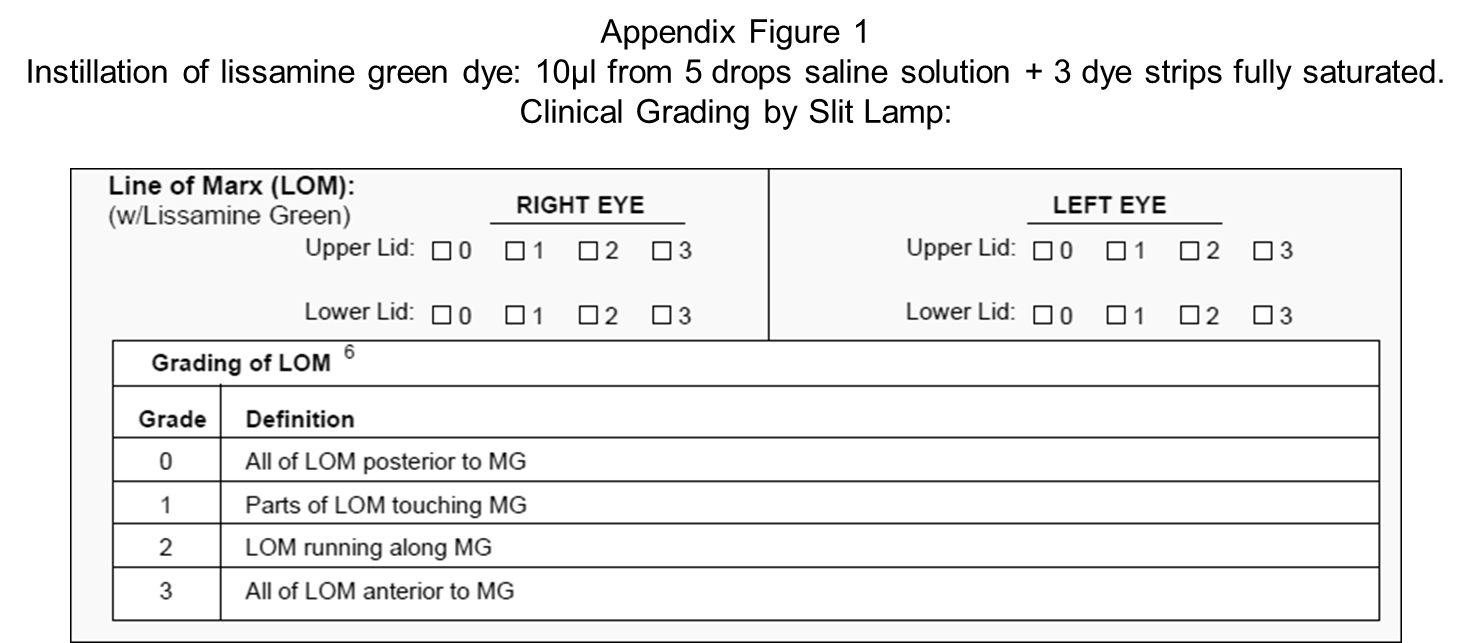


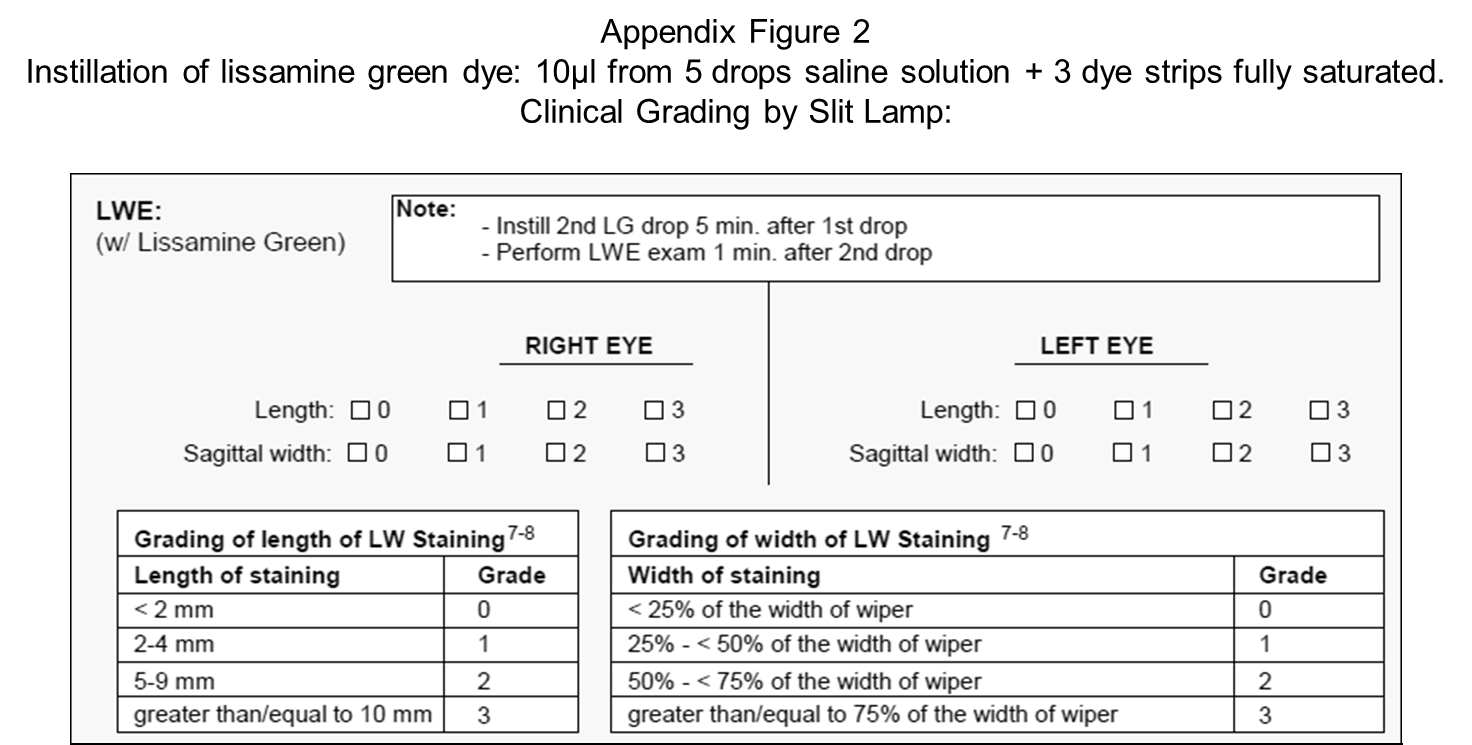


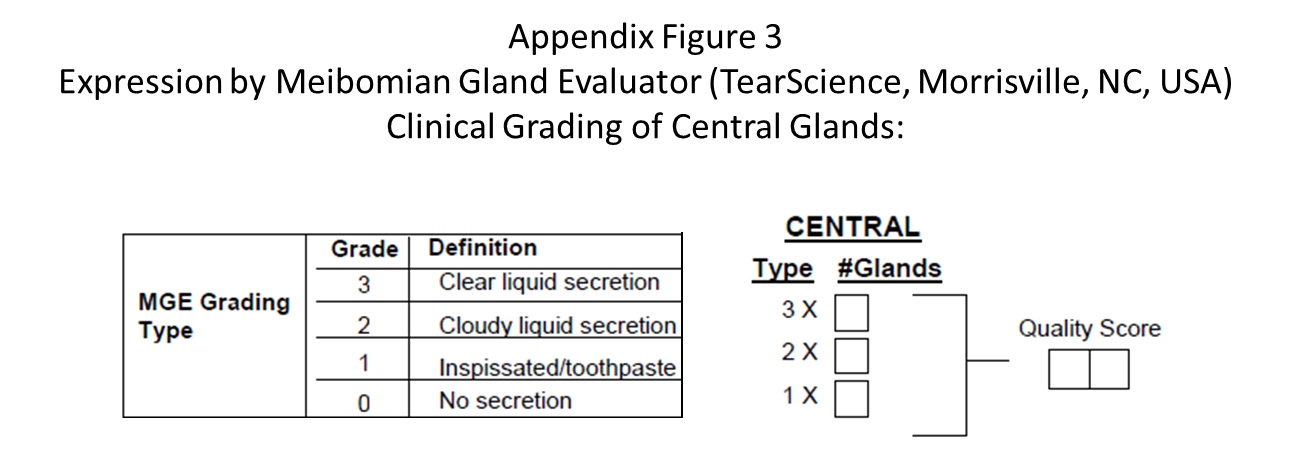

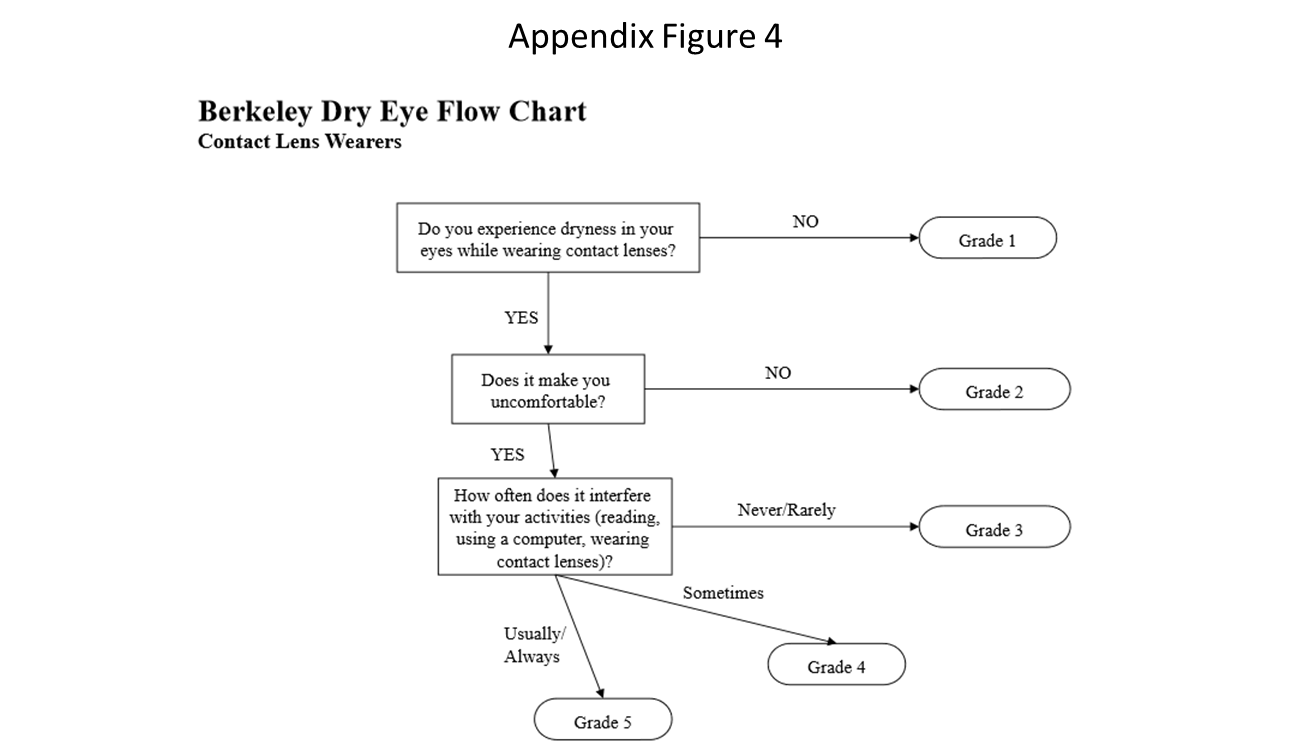


For the DEFC, the questionnaire administrator instructs the respondent to consider how their eyes have felt over the past week. To distinguish contact lens-induced dry eye from physiological dry eye for contact lens wearers, the DEFC is repeated with the first item phrased as “Do you experience dryness in your eyes after removing your contact lenses?”. For the non-contact lens wearer version of the DEFC, the first and last items are phrased without reference to wearing contact lenses.

A focus group consisting of the lead clinical investigator (MCL) and three experienced optometrists examined previously collected subject records and made independent diagnoses of Meibomian gland dysfunction, blepharitis, and lagophthalmos. After multiple rounds of record reviews, diagnoses, and discussions with the lead clinical investigator, the above standardized diagnostic procedures and criteria were established in order to calibrate all observers to the same criteria.
